# Cannabidiol combined with GABAergic drugs but not with sodium channel blockers prevents the development of drug-resistance seizures in a preclinical model

**DOI:** 10.3389/fphar.2025.1644018

**Published:** 2025-09-19

**Authors:** Monserrat Fuentes-Mejia, Maximiliano J. Fallico, Alan Talevi, Luciana Gavernet, Sandra A. Orozco-Suárez, Luisa Rocha

**Affiliations:** ^1^ Pharmacobiology Department, Center for Research and Advanced Studies of the National Polytechnic Institute, Mexico City, Mexico; ^2^ Laboratory of Bioactive Compounds Research and Development (LIDeB), Department of Biological Sciences, Faculty of Exact Sciences, CCT La Plata, Consejo Nacional de Investigaciones Científicas y Técnicas (CONICET), National University of La Plata (UNLP), La Plata, Argentina; ^3^ Neurological Diseases Medical Research Unit, Specialty Hospital, “Dr. Bernardo Sepúlveda”, National Medical Center XXI, Century, Mexican Social Security Institute (IMSS), Mexico City, Mexico

**Keywords:** cannabidiol, drug-resistance, seizures, GABAergic drugs, sodium channel blockers

## Abstract

Drug resistance affects 30% of patients with epilepsy. Cannabidiol (CBD) decreases the expression of drug-resistant seizures in specific syndromes. However, it is unknown if CBD prevents the development of drug-resistant condition in epilepsy. This research was conducted to investigate if subchronic administration of CBD with sodium channel blockers modifies the mortality associated with clonic-tonic seizures and the development of the drug-resistant phenotype induced by subchronic administration of 3-mercaptopropionic acid (3-MP) in rats. These effects were compared with those elicited by antiseizure medications acting on the GABA_A_ receptors. Male Wistar rats were used to evaluate CBD combined with different antiseizure medications (phenobarbital, diazepam, valproic acid, lamotrigine and oxcarbazepine) during the repetitive administration of 3-MP. The mortality rate and development of drug-resistant seizures were estimated. Computational experiments explored interactions between CBD and sodium channel blockers in the NaV1.7 receptor. Subchronic administration of CBD alone did not modify neither the mortality rate nor the development of drug-resistant seizures. CBD combined with phenobarbital or diazepam reduced the mortality rate and prevalence of drug-resistant seizures. In contrast, coadministration of CBD with valproic acid or lamotrigine did not modify neither the mortality rate nor the expression of drug-resistant seizures. Contrariwise, combining CBD with oxcarbazepine at ED_50_ increases the incidence of drug-resistant seizures. Computational experiments suggested that CBD acting on NaV1.7 interferes with the action of sodium channel blockers and precludes their inhibitory effects. Our results indicate that repeated administration of CBD with GABAergic antiseizure medications, but not sodium channel blockers, decreases the mortality and prevents the development of the drug-resistant phenotype induced by repeatedly provoked severe seizures.

## 1 Introduction

Cannabidiol (CBD), the main non-psychoactive component of *Cannabis* plants (Russo, 2017), is known to reduce seizure activity in humans and experimental models (Silvestro et al., 2019). Used as adjunctive therapy, CBD reduces the frequency and severity of seizures associated with Dravet and Lennox-Gastaut syndromes, and tuberous sclerosis (Maa and Figi, 2014; Thiele et al., 2018; Lazaridis et al., 2019). Studies support that the coadministration of CBD with antiseizure medications (ASMs) decreases the frequency and severity of seizures associated with drug-resistant temporal lobe epilepsy (Cunha et al., 1980). This effect is also evident in experimental models of temporal lobe epilepsy (Khan et al., 2018; Patra et al., 2019). On the other hand, a previous study indicated that the administration of CBD alone did not modify the mortality rate in a mouse model of Dravet syndrome (Anderson et al., 2020).

In particular, it has been reported that the antiseizure effects of GABAergic ASMs are increased when chronically coadministered with CBD in patients (Devinsky et al., 2016; Hess et al., 2016; Anderson et al., 2019). Frías-Soria et al. (2021) found that the subchronic administration of CBD combined with phenobarbital during the repetitive induction of seizures with 3-mercaptopropionic acid reduced the ictal severity in rats. This effect is explained by the action of CBD as a positive allosteric modulator of the GABA_A_ receptor (Bakas et al., 2017). The repetitive administration of CBD during hippocampal kindling decreases the severity of epileptogenesis, an effect that is accentuated when coadministered with midazolam and ganaxolone, which act on GABA_A_ receptors, but not with tiagabine, which blocks GABA uptake (Reddy et al., 2023). This study suggests that concomitant use of CBD with some ASMs acting on GABA_A_ receptors could produce disease-modifying effects.

In contrast, the results obtained with the combination of acute and chronic CBD with sodium channel blockers are controversial. *In vitro* and structural studies showed that CBD interacts with sodium channels at nanomolar concentrations producing a direct stabilization of the inactivated state (Huang et al., 2023a). An *in vitro* study revealed that CBD reduces neuronal excitability of human layer V pyramidal neurons obtained from epilepsy surgery of patients with drug-resistant epilepsy. This effect was associated with the modulatory action of CBD on the voltage-sensitive Na + currents underlying spike generation in human epileptic neocortex (Martinez-Rojas et al., 2025). On the other hand, Frías-Soria et al. (2021) described that the repetitive coadministration of CBD with phenytoin, a sodium channel blocker, increased the prevalence of status epilepticus in rats during the sub-chronic administration of 3-mercaptopropionic acid (3-MP). Using the maximal electroshock- and 6 Hz-induced seizures in mice, Socała et al. (2019) found that CBD increased the efficacy of topiramate and oxcarbazepine and did not affect the effect of lamotrigine and lacosamide. This study also revealed that CBD reduced the antiseizure efficacy of levetiracetam, an effect that the authors explained via a pharmacodynamic interaction between the two drugs.

According to these studies, it is possible to hypothesize that the reduced antiseizure effect of some sodium channel blockers when coadministered with CBD results from their interaction in the same binding sites. The investigation of this issue is essential because the coadministration of CBD with specific sodium channel blockers can exacerbate the convulsive activity in syndromes with severe seizures. The present study focused on examining if subchronic administration of CBD with sodium channel blockers may modify the mortality rate during the repetitive induction of clonic-tonic seizures and the development of the drug-resistant seizures in a preclinical model. The effects obtained were compared with those found with ASMs acting on the GABA_A_ receptors. We analyze the effects of CBD combined with the ASMs at ED_30_ and ED_50_. This strategy allowed us to investigate if CBD enhances the inhibitory effects of the ASMs at low doses (ED_30_), a situation that may reduce the incidence of side effects. Available structural data and docking simulations permitted us to propose an explanation, at the molecular level, for the results found.

We used the administration of 3-MP that induces severe seizures characterized by a violent running fit followed by clonic-tonic convulsions. 3-MP produces seizures as consequence of lower GABAergic neurotransmission as result of inhibition of the glutamic acid decarboxylase enzyme (GAD) and activation of GABA-α-oxoglutarate aminotransferase (GABA-T). 3-MP-induced seizures cause damage in several brain regions (O’Connell et al., 1988). Notably, this model results in damage of the substantia nigra—a key endogenous seizure-suppressing area (Towfighi et al., 1989; Velíšková and Moshé, 2006). Phenobarbital, phenytoin, diazepam, carbamazepine and sodium valproate are effective to prevent 3-MP-induced acute convulsions (Löscher, 1979). Repeated administration of 3-MP progressively enhances the seizure severity (Frías-Soria et al., 2021) and develops resistance to ASMs (Enrique et al., 2017; Pérez-Pérez et al., 2021) associated with overexpression of brain P-glycoprotein (Lazarowski et al., 2004; Rosillo-de la Torre et al., 2015; Enrique et al., 2017).

## 2 Materials and methods

### 2.1 Animals

Wistar male rats (n = 766) weighing 250–300 g were used. They were kept under 20 °C–22 °C, humidity levels of 50%–60% and 12-h light/dark cycle. Food and water were provided *ad libitum*. Animals were habituated to manipulation by receiving a daily administration of saline solution (1 mL/kg, i.p.) during 7 days. Habituation was carried out to avoid the influence of stress in the results obtained because it mitigates changes on blood pressure, heart rate (Bonnichsen et al., 2005), drug side effects (Abbott et al., 2006), nociception (Smythe et al., 1994) and sensorimotor gating (Santos-Carrasco et al., 2025) induce during the experimental procedure. The present study adhered to the Mexican Official Standard (NOM-062-ZOO-1999) and received approval from the Ethics Committee of the Center for Research and Advanced Studies (CICUAL 0394-24).

### 2.2 Estimation of the ED_30_ and ED_50_ to prevent clonic-tonic seizures induced by an acute dose of 3-MP

Initially, a group of animals (n = 300) previously habituated to manipulation (see Section 2.1) was used to estimate the ED_30_ and ED_50_ of phenobarbital, diazepam, valproic acid, lamotrigine, and oxcarbazepine to prevent severe clonic-tonic seizures (wild jump, running and clonic-tonic seizures) induced by an acute administration of 3-MP. For this purpose, animals pretreated with one ASM (n = 10 per dose per ASM) were treated with 3-MP (37.5 mg/kg i.p.). The interval between the administration of the ASMs and 3-MP was 1 h, except for diazepam (2 h). Thereafter, the expression of severe generalized seizures was estimated during 30 min. To estimate the dose-response curve, each ASM had at least six doses evaluated (dose ranges for each drug are presented in Table 1). The data were analyzed using nonlinear regression to determine the ED_30_ and ED_50_ of each ASM (Motulsky and Christopoulos, 2003).

**TABLE 1:** Doses and route of administration of the different antiseizure medications to identify their ED_30_ and ED_50_ to prevent clonic-tonic seizures induced by 3-mercaptopropionic acid (37.5 mg/kg, i.p.).

Antiseizure medications (range of doses and route of administration)	Dose (mg/kg)	
	ED_30_	ED_50_
Diazepam (0.47–31.6 mg/kg i.m.)	0.7	1
Phenobarbital (1–39.8 mg/kg i.p.)	12	15
Valproic Acid (1–316 mg/kg i.p.)	38	46
Lamotrigine (0.31–100 mg/kg i.p.)	6	10
Oxcarbazepine (0.31–100 mg/kg i.p.)	8	12

ED_30_, effective dose 30; ED_50_, effective dose 50.

### 2.3 Evaluation of the effects of the repetitive administration of ASMs combined with CBD on sensorimotor function

A second experiment focused on determining if repetitive administration of CBD combined with ASMs induces side effects on sensorimotor function that may confound anticonvulsant effects. For this experiment, rats previously habituated (see Section 2.1) received CBD (200 mg/kg p.o.), or coconut oil (CCO, 9.5 mL/kg p.o.). One hour later, they received an ASM at ED_30_ or ED_50_, or vehicle (saline solution 1 mL/kg i.p.). We used 3 rats per each treatment (a total of 66 rats). This procedure was repeated every 12 h during 10 trials. The sensorimotor function was evaluated under basal conditions (without treatment), and after the first, fifth, and 10th trials as well as 48 h after the last administration. The interval between the administration of the ASM and the evaluation of the sensorimotor function was 1 h except for diazepam (2 h).

The sensorimotor function was estimated using Neuroscore (Pierce et al., 1998), a battery of tests that evaluates different motor responses such as the ability to remain on an inclined surface at different angles (35°–75°) in both vertical and lateral positions (left (L) and right (R)), hind and forelimb contraflexion (L-R) during tail suspension, and resistance to lateral displacement (L-R). A score of 26–28 indicates normal function, whereas lower scores suggest sensorimotor impairment. The sensorimotor results were assessed by two-way ANOVA *post hoc* Tukey test.

### 2.4 Evaluation of the development of drug-resistant seizures in animals receiving the coadministration of CBD and ASMs

This experiment was designed to determine if the administration of CBD in combination with ASMs at ED_30_ or ED_50_ could have an impact on the mortality rate and development of drug-resistant seizures as consequence of the repetitive administration of 3-MP. Remarkably, repetitive administration of 3-MP is known to induce over-expression of P-glycoprotein in the brain and drug-resistance to phenytoin and phenobarbital in rodents (Lazarowski et al., 2004; Enrique et al., 2017; Pérez-Pérez et al., 2021).

For the present study, we used the protocol previously reported by Pérez-Pérez et al. (2021) to induce drug-resistant seizures. Rats (n = 400) were habituated as previously described (see Section 2.1). One day after finishing the habituation, a group of animals was used for evaluating the mortality rate and the expression of phenobarbital-resistant seizures after receiving the subchronic coadministration of CBD plus phenobarbital. For this purpose, different experimental groups were designed to receive one of the following treatments:

a. CBD plus phenobarbital at ED_50_. CBD (200 mg/kg p.o.) was delivered 1 h before phenobarbital. 1 h after phenobarbital administration, 3-MP was applied at an initial dose of 30 mg/kg, i.p. This procedure was repeated every 12 h during 10 trials. The 3-MP dose was gradually increased through the experimental procedure up to 37.5 mg/kg to maintain the induction of severe clonic-tonic seizures (Enrique et al., 2017). The mortality rate was evaluated during the repetitive administration of 3-MP. 12 h after the last 3-MP administration, an assay focused on identifying animals with phenobarbital-resistant seizures was carried out in 10 surviving rats. The assay consisted in the administration of phenobarbital and 60 min later, a proconvulsant dose of 3-MP (37.5 mg/kg). Animals showing clonic-tonic seizures during this assay were considered resistant to phenobarbital.
b. CBD plus phenobarbital at ED_30_. Rats were manipulated as described for the treatment with CBD plus phenobarbital at ED_50_, except that phenobarbital was delivered at ED_30_.
c. Coconut oil plus phenobarbital at ED_50_. Animals were manipulated as indicated for the treatment with CBD plus phenobarbital at ED_50_, but receiving coconut oil instead of CBD.
d. Coconut oil plus phenobarbital at ED_30_. Rats were manipulated as indicated for the treatment with coconut oil plus phenobarbital at ED_50_, but receiving phenobarbital at ED_30_.
e. CBD plus vehicle. Animals were manipulated as specified for the treatment with CBD plus phenobarbital at ED_50_, except that vehicle was applied instead of phenobarbital.
f. Coconut oil plus vehicle. Rats were manipulated as indicated for the treatment with CBD plus vehicle, but receiving coconut oil instead of CBD.

Similar treatments were designed with diazepam, valproic acid, lamotrigine and oxcarbazepine to identify rats with drug-resistant seizures to these ASMs.

The total number of animals used for each treatment depended on the mortality rate (see Table 3). The mortality rate during the repetitive administration of 3-MP and the prevalence of animals with drug-resistant seizures at the end of the experimental protocol were analyzed employing Fisher’s exact test (one-side) and using GraphPad Prism version 8 for Windows (GraphPad Software, Inc.). A p < 0.05 was considered significant.

### 2.5 Computational studies

The interactions between sodium channels and CBD, lamotrigine and oxcarbazepine were studied via docking simulations. Autodock-GPU and Autodock Vina were considered to identify which software was best suited for this particular system.

**FIGURE 1:**
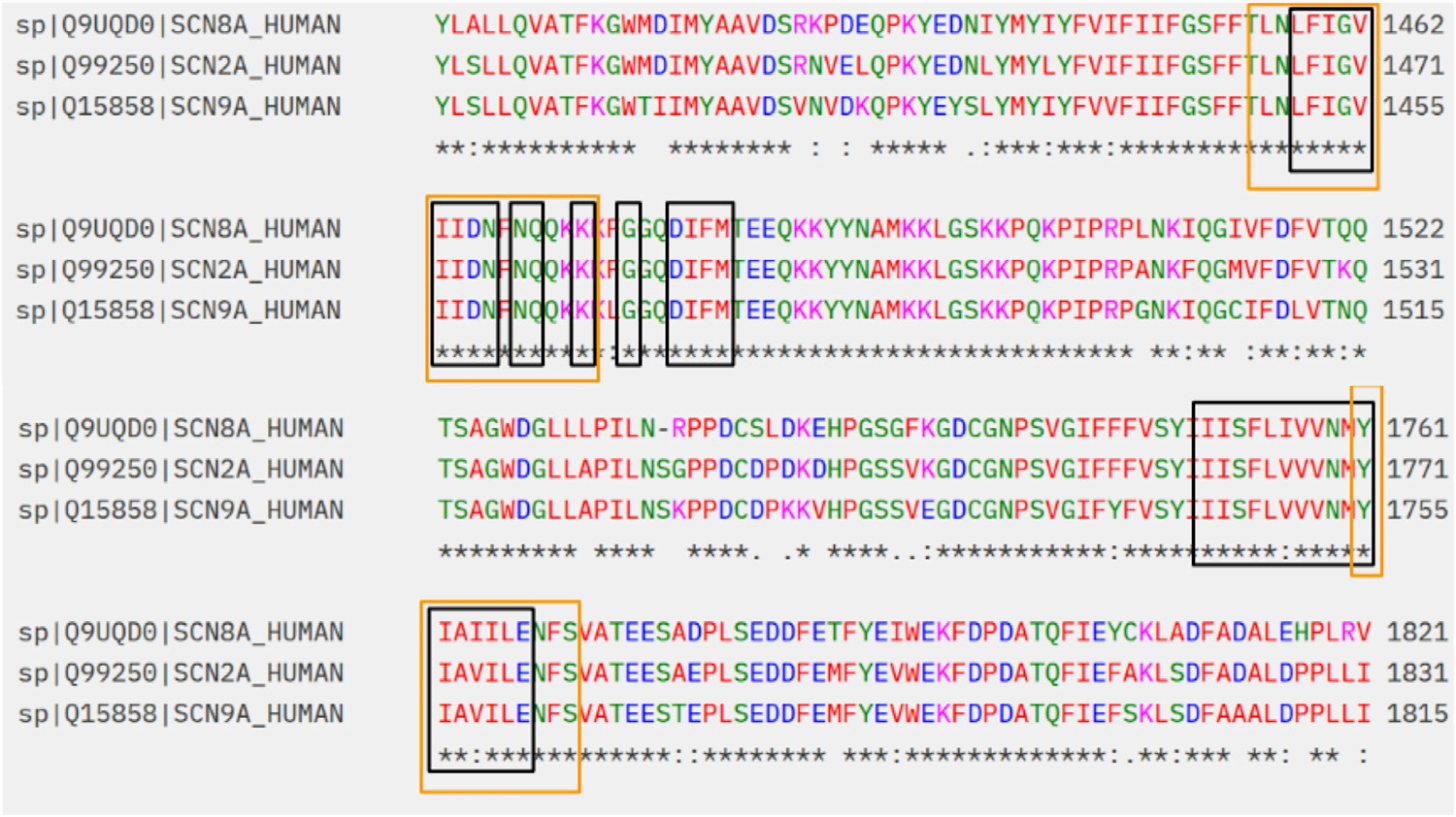
Extract of the sequence alignment between NaV1.7, NaV1.6 and NaV1.2. The amino acids that belong to the I-site and BIG site regions are framed in black and orange, respectively.

The docking regions were defined from recent structural studies based on cryo-EM structures of human NaV1.7 by Huang et al. (2023a) and Wu et al. (2023), who described two binding regions apart from the central cavity of the pore domain. To evaluate whether the binding modes in NaV1.7 of CBD and classic antiseizure medications based on blockade of sodium currents may be reasonably extrapolated to other channel subtypes, a structural comparison between the binding regions of NaV1.7 and the isoforms associated with seizure control (NaV1.2 and NaV1.6) was performed by sequence-structure alignment tools in UCSF Chimera, using Needleman-Wunsch algorithm with BLOSUM-62 matrix for the alignment. The alpha subunits were retrieved from PDB (PDB-IDs 8G1A for NAV1.7, 6J8E for NaV1.2, and 8FHD for NaV1.6). The analysis was restricted to a radius of 10 Å from the centers of the binding regions, which were defined by the positions of the CBD (I-site) and carbamazepine (BIG-site) in the experimental structures. The high levels of sequence identity in the α-subunit for all isoforms, and in the BIG and I-sites (>80%, Figure 1) supported the extrapolation of the observations reported for this receptor to epilepsy-related NaV isoforms.

## 3 Results

### 3.1 Estimation of the ED_30_ and ED_50_ to prevent clonic-tonic seizures induced by an acute dose of 3-mercaptopropionic acid (3-MP)

The control group receiving vehicle before the acute administration of 3-MP showed a prevalence of 100% of severe clonic-tonic seizures. The dose-response curve obtained from the different doses evaluated from each ASM allowed the estimation of the ED_30_ and ED_50_ to prevent severe clonic-tonic seizures (Table 1).

### 3.2 The repetitive administration of CBD plus ASMs at ED_30_ or ED_50_ does not modify the sensorimotor function

Rats receiving the repetitive administration of coconut oil plus vehicle showed an average Neuroscore index of 27.06 ± 0.18, indicating normal sensorimotor function. Similarly, the repetitive administration of CBD alone or in combination with the different ASMs at ED_30_ or ED_50_ also yield a Neuroscore index in the range of 26–28. These results support that the repetitive coadministration of CBD with the different ASMs at ED_30_ or ED_50_ did not induce sensorimotor side effects (Table 2).

**TABLE 2:** Evaluation of the sensorimotor function with Neuroscore during the repetitive administration of CBD combined with ASMs in normal rats.

Treatments		Number of administrations				
		Basal	1	5	10	48 h after 10
CCO + VHE (n = 3)		27.63 ± 0.57	26.72 ± 0.69	27.06 ± 0.41	27.39 ± 0.53	27.50 ± 0.86
CBD + VHE (n = 3)		27.67 ± 0.52	27.06 ± 0.91	26.57 ± 0.83	26.78 ± 1.01	26.11 ± 1.45
ED_30_	CCO + VPA (n = 3)	26.83 ± 0.44	27.50 ± 0.49	27.17 ± 0.31	26.28 ± 0.25	27.02 ± 0.76
	CBD + VPA (n = 3)	27.33 ± 0.28	26.44 ± 0.51	27.11 ± 0.09	26.50 ± 0.43	27.05 ± 0.12
	CCO + LMG (n = 3)	27.59 ± 0.31	27.13 ± 0.08	26.68 ± 0.65	27.16 ± 0.58	27.51 ± 0.62
	CBD + LMG (n = 3)	27.22 ± 0.63	27.36 ± 0.19	27.52 ± 0.56	26.65 ± 0.41	27.24 ± 0.23
	CCO + OXC (n = 3)	27.33 ± 0.52	27.13 ± 0.43	27.28 ± 0.62	27.64 ± 0.37	26.36 ± 0.59
	CBD + OXC (n = 3)	27.48 ± 0.35	27.07 ± 0.18	26.48 ± 0.77	26.89 ± 0.61	26.85 ± 0.46
	CCO + DZP (n = 3)	26.79 ± 0.92	27.19 ± 0.62	27.32 ± 0.53	27.41 ± 1.23	27.59 ± 0.35
	CBD + DZP (n = 3)	27.42 ± 1.14	26.85 ± 0.97	26.99 ± 0.76	27.32 ± 0.45	26.73 ± 1.05
	CCO + PB (n = 3)	26.78 ± 0.69	26.89 ± 0.67	26.39 ± 1.01	27.06 ± 1.05	26.44 ± 1.34
	CBD + PB (n = 3)	27.37 ± 0.39	27.65 ± 0.82	26.44 ± 0.91	26.83 ± 0.74	26.87 ± 0.57
ED_50_	CCO + VPA (n = 3)	26.86±	27.41 ± 0.36	27.09 ± 0.18	27.45 ± 1.15	27.51 ± 0.96
	CBD + VPA (n = 3)	27.61 ± 1.12	27.61 ± 0.36	27.27 ± 0	27.38 ± 0.29	27.47 ± 1.01
	CCO + LMG (n = 3)	26.72 ± 0.33	27.11 ± 0.42	27.22 ± 0.11	26.72 ± 0.67	27.03 ± 0.80
	CBD + LMG (n = 3)	26.83 ± 0.25	27.21 ± 0.52	27.15 ± 0.46	27.43 ± 0.24	27.10 ± 0.47
	CCO + OXC (n = 3)	27.54 ± 0.63	27.57 ± 0.09	26.61 ± 0.42	26.45 ± 0.35	27.02 ± 0.32
	CBD + OXC (n = 3)	26.57 ± 0.17	27.40 ± 0.63	27.26 ± 0.21	26.56 ± 0.65	27.49 ± 0.48
	CCO + DZP (n = 3)	27.39 ± 0.19	27.12 ± 0.49	26.76 ± 0.56	27.16 ± 0.41	27.54 ± 0.63
	CBD + DZP (n = 3)	27.45 ± 0.32	27.69 ± 0.23	27.11 ± 0.61	27.17 ± 0.26	27.06 ± 0.54
	CCO + PB (n = 3)	26.86 ± 0.55	27.33 ± 0.81	27.68 ± 0.25	27.46 ± 0.17	27.21 ± 0.92
	CBD + PB (n = 3)	27.07 ± 0.19	26.96 ± 0.72	27.07 ± 0.45	27.41 ± 0.23	26.81 ± 0.98

The values represent the mean ± SD of the scores obtained with the Neuroscore battery. ASM, antiseizure medication; CCO, coconut oil; CBD, cannabidiol; DZP, diazepam; ED_30_, effective dose 30; ED_50_, effective dose 50; LMG, lamotrigine; OXC, oxcarbazepine; PB, phenobarbital; VHE, vehicle; VPA, valproic acid.

### 3.3 CBD is effective in reducing drug-resistant seizures when combined with GABAergic drugs but not with sodium channel blockers

Experimental groups receiving coconut oil plus vehicle during the repetitive administration of 3-MP showed an elevated mortality rate. In contrast, coadministration of CBD and phenobarbital at ED_30_ or ED_50_ during the repetitive 3-MP administration resulted in 0% of mortality (p = 0.0163 vs coconut oil plus vehicle for both doses). Similarly, coadministration of CBD and diazepam at ED_50_ avoided the mortality (0%, p = 0.0433 vs. coconut oil plus vehicle). On the other hand, a high mortality rate (41%) was obtained when animals received the subchronic administration of CBD plus oxcarbazepine at ED_50_. Other treatments did not significantly modify the mortality rate during the repetitive administration of 3-MP (Table 3).

**TABLE 3:** Effects of the different treatments applied on the mortality rate during the subchronic administration of 3-mercaptopropionic acid.

ASM evaluated	Mortality rate as consequence of the subchronic administration of CBD plus ASMs during repetitive 3-MP			
Phenobarbital ED_30_ (12 mg/kg)	CCO + VHE 50% (10/20)	CBD + VHE 41% (7/17)	CCO + PB 29% (4/14)	CBD + PB 0% (0/10)*
ED_50_ (15 mg/kg)	41% (7/17)	37% (6/16)	23% (3/13)	0% (0/10)*
Diazepam ED_30_ (0.7 mg/kg)	CCO + VHE 50% (10/20)	CBD + VHE 37% (6/16)	CCO + DZP 17% (2/12)	CBD + DZP 9% (1/11)
ED_50_ (1 mg/kg)	41% (7/17)	33% (5/15)	17% (2/12)	0%* (0/10)
Valproic acid ED_30_ (38 mg/kg)	CCO + VHE 50% (10/20)	CBD + VHE 41% (7/17)	CCO + VPA 37% (6/16)	CBD + VPA 29% (4/14)
ED_50_ (46 mg/kg)	50% (10/20)	37% (6/16)	29% (4/14)	17% (2/12)
Lamotrigine ED_30_ (6 mg/kg)	CCO + VHE 50% (10/20)	CBD + VHE 50% (10/20)	CCO + LMG 23% (3/13)	CBD + LMG 33% (5/15)
ED_50_ (10 mg/kg)	44% (8/18)	37% (6/16)	33% (5/15)	41% (7/17)
Oxcarbazepine ED_30_ (8 mg/kg)	CCO + VHE 50% (10/20)	CBD + VHE 50% (10/20)	CCO + OXC 29% (4/14)	CBD + OXC 44% (8/18)
ED_50_ (12 mg/kg)	44% (8/18)	33% (5/15)	17% (2/12)	41% (7/17)

The values represent the percentage (death/total used) of rats that died during the repetitive administration of the different treatments. ASM, antiseizure medication; CCO, coconut oil; CBD, cannabidiol; DZP, diazepam; ED_30_, effective dose 30; ED_50_, effective dose 50; LMG, lamotrigine; OXC, oxcarbazepine; PB, phenobarbital; VHE, vehicle; VPA, valproic acid.

*p < 0.05, **p < 0.01, ***p < 0.0001 vs CCO + VHE treatment; ^$^p < 0.05 vs CBD + VHE treatment; &p < 0.05 vs CCO + DZP treatment; ^##^p < 0.001 vs CCO + PB treatment.

The drug-resistant assay carried out by the end of the experimental procedure revealed the high prevalence of drug-resistant seizures to the different ASMs in the experimental groups receiving repetitive coconut oil plus vehicle. Low prevalence of animals with phenobarbital-resistant seizures was found when applied CBD plus phenobarbital at ED_30_ (p = 0.005 vs coconut oil plus phenobarbital at ED_30_) or ED_50_ (p = 0.009 vs. coconut oil plus phenobarbital at ED_50_). Similarly, coadministration of CBD and diazepam at ED_50_ reduced the prevalence of diazepam-resistant seizures (p = 0.0286 vs. coconut oil plus diazepam at ED_50_). Contrariwise, high prevalence (100%) of resistant seizures to oxcarbazepine (p = 0.0163 vs. coconut oil plus vehicle) was observed when animals received the subchronic administration of CBD plus oxcarbazepine at ED_50_. No significant changes were detected with other treatments (see Table 4).

**TABLE 4:** Effects of the different treatments applied on the expression of drug-resistant seizures following the subchronic administration of 3-mercaptopropionic acid.

ASM evaluated during the drug-resistant assay		Animals with drug-resistant seizures as consequence of the subchronic administration of CBD plus ASMs during repetitive 3-MP		
Phenobarbital ED_30_ (12 mg/kg)	CCO + VHE 90% (9/10)	CBD + VHE 40% (4/10)	CCO + PB 60% (6/10)	CBD + PB 0% (0/10) ***^$##^
ED_50_ (15 mg/kg)	70% (7/10)	20% (2/10)	70% (7/10)	10% (1/10) **^##^
Diazepam ED_30_ (0.7 mg/kg)	CCO + VHE 90% (9/10)	CBD + VHE 50% (5/10)	CCO + DZP 50% (5/10)	CBD + DZP 30% (3/10) **
ED_50_ (1 mg/kg)	90% (9/10)	60% (6/10)	60% (6/10)	10% (1/10)***^$&^
Valproic acid ED_30_ (38 mg/kg)	CCO + VHE 60% (6/10)	CBD + VHE 60% (6/10)	CCO + VPA 30% (3/10)	CBD + VPA 40% (4/10)
ED_50_ (46 mg/kg)	50% (5/10)	60% (6/10)	60% (6/10)	70% (7/10)
Lamotrigine ED_30_ (6 mg/kg)	CCO + VHE 80% (8/10)	CBD + VHE 60% (6/10)	CCO + LMG 30% (3/10)	CBD + LMG 40% (4/10)
ED_50_ (10 mg/kg)	70% (7/10)	50% (5/10)	40% (4/10)	50% (5/10)
Oxcarbazepine ED_30_ (8 mg/kg)	CCO + VHE 60% (6/10)	CBD + VHE 80% (8/10)	CCO + OXC 60% (6/10)	CBD + OXC 80% (8/10)
ED_50_ (12 mg/kg)	50% (5/10)	60% (6/10)	70% (7/10)	100% (10/10) *^$^

Values represent the percentage of rats with drug-resistant seizures. In parenthesis, it is indicated the number of rats with drug-resistant seizures/total used per treatment. ASM, antiseizure medication; CCO, coconut oil; CBD, cannabidiol; DZP, diazepam; ED_30_, effective dose 30; ED_50_, effective dose 50; LMG, lamotrigine; OXC, oxcarbazepine; PB, phenobarbital; VHE, vehicle; VPA, valproic acid.

*p < 0.05, **p < 0.01, ***p < 0.0001 vs CCO + VHE treatment; ^$^p < 0.05 vs CBD + VHE treatment; &p < 0.05 vs CCO + DZP treatment; ^##^p < 0.001 vs CCO + PB treatment.

### 3.4 Computational studies

**FIGURE 2:**
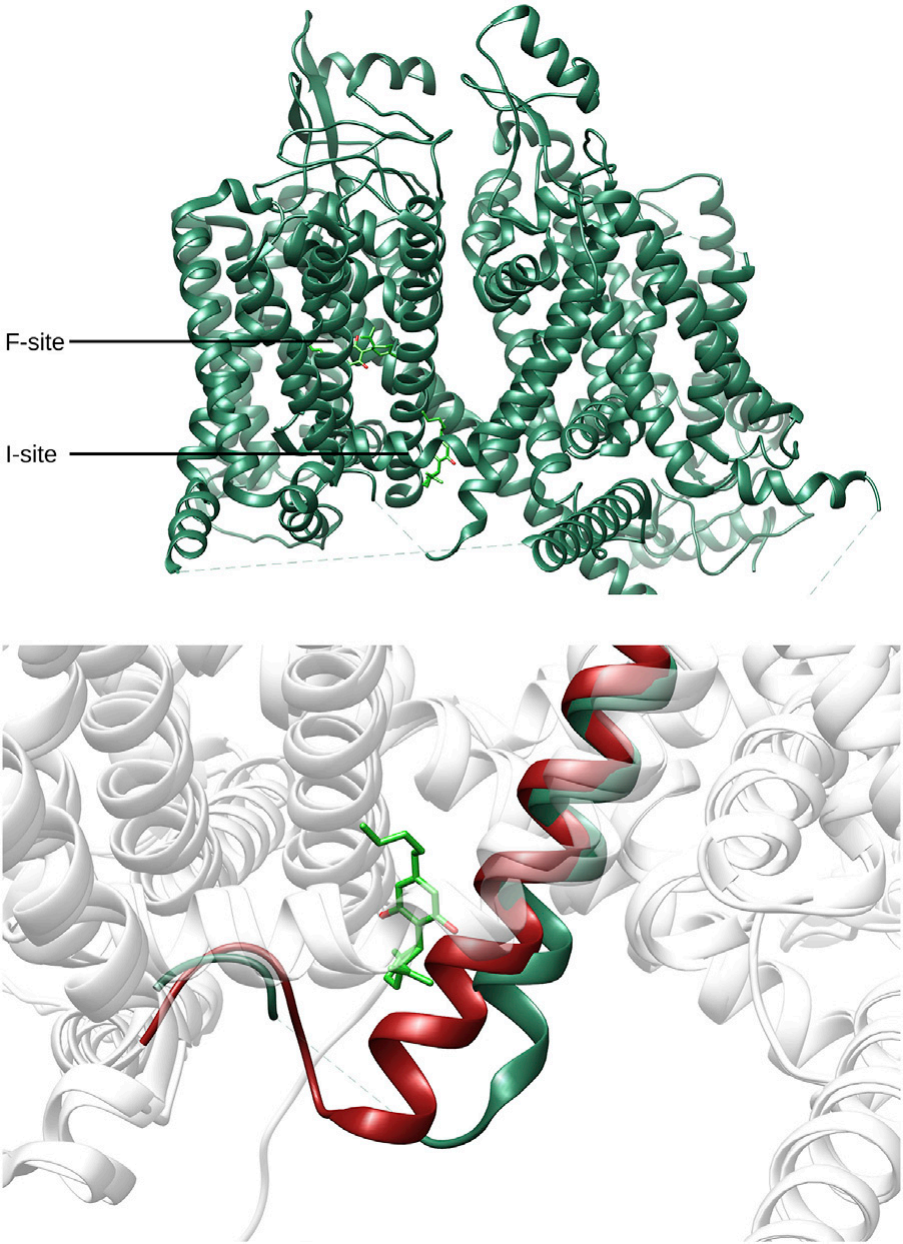
Top: Location of the Fenestration site (F-site) and Isoleucine-Phenylalanine-Methionine site (I-site) found for CBD (lime green) in NaV1.7 (PDB-ID: 8G1A, green). Bottom: Comparison between the I-site conformation found for CBD and the corresponding region found in apo conformation of NaV1.7 (PDB-ID: 7W9K, red).

**FIGURE 3:**
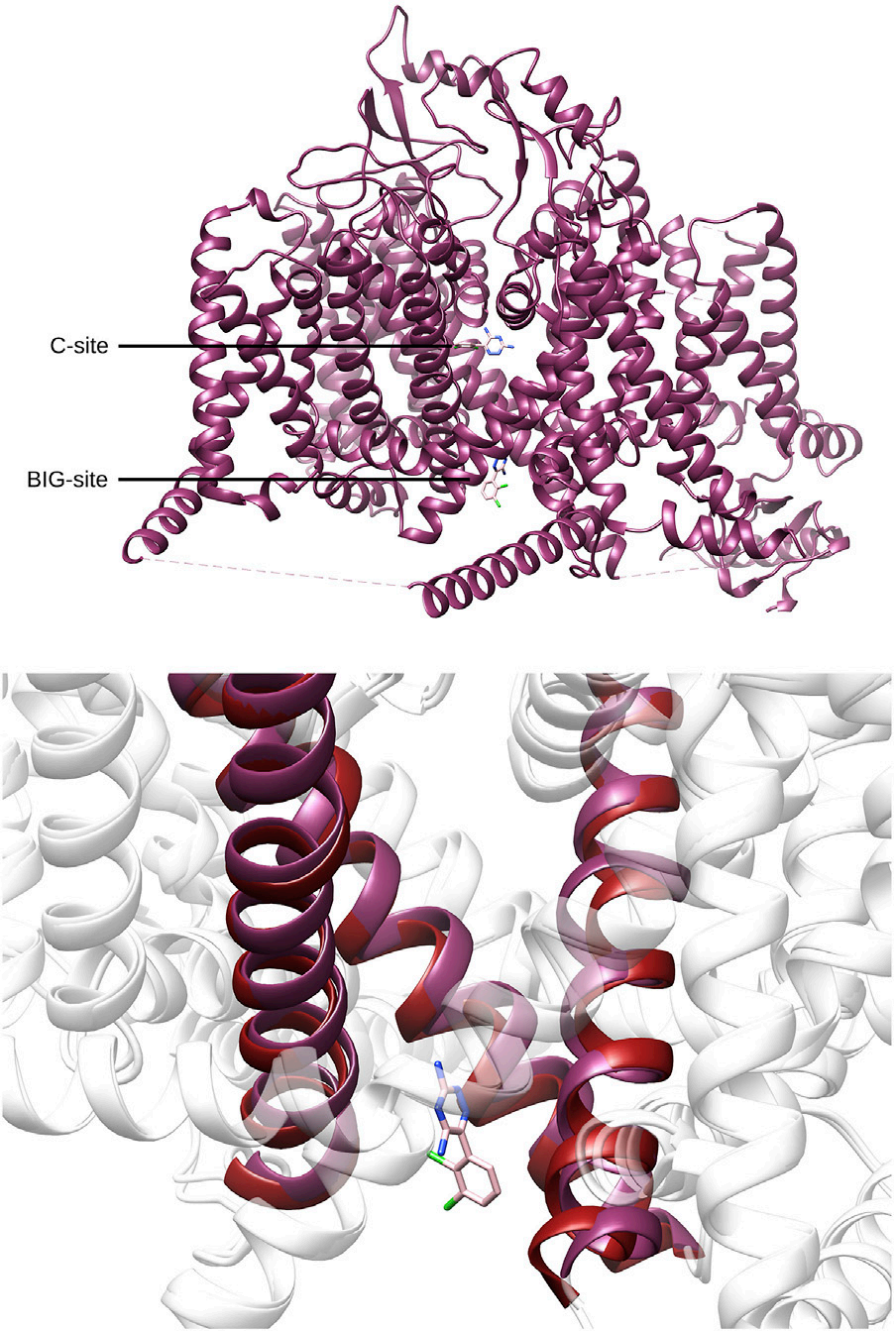
Top. Location of the central cavity binding site (C-site) and the region beneath the intracellular gate (BIG site) found for lamotrigine (pink) in NaV1.7(PDB-ID: 8THH, magenta). Bottom. Comparison between the BIG-site conformation found for lamotrigine and the corresponding region found in apo conformation of NaV1.7 (PDB-ID: 7W9K, red).

Recent studies (Huang et al., 2023b; Huang et al., 2023a; Wu et al., 2023) provided structural information about the interaction of CBD and different anesthetic drugs and ASMs (including carbamazepine and lamotrigine) with the NaV1.7 isoform of human sodium channels. They identified two binding sites for CBD, one in a fenestration in the upper pore region (IV-I fenestration, F-site) and the other close to the Isoleucine-Phenylalanine-Methionine (IFM) motif near the intracellular linker between domains III and IV (I-site). We found that CBD induces important conformational changes in the I-site, mainly in the S6III region, which stabilizes the inactivated state of the channel via an allosteric effect (Figure 2). In the case of lamotrigine, a dual-pocket inhibition was proposed (Figure 3), with one binding site in the central cavity of the pore domain (C-site), and a second one beneath the intracellular gate (BIG site). This same binding region was previously identified for carbamazepine (Wu et al., 2023).

**FIGURE 4:**
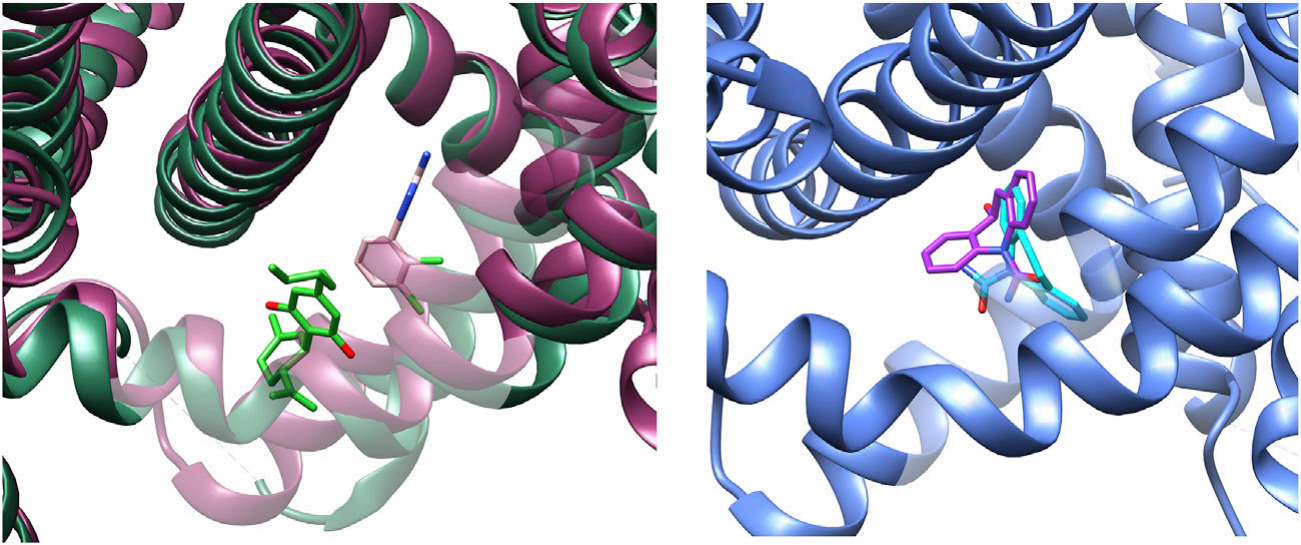
Left: Experimental conformations of the binding sites found for NaV1.7 in presence of CBD (I-site) and lamotrigine (BIG site). CBD (lime green) and lamotrigine (pink) structures are retained as reference. Right: Docking pose prediction for S-MDH into the BIG site (PDB-ID: 8S9C, blue). S-MDH prediction is represented in purple color, while experimental carbamazepine is represented in light blue as reference.

Binding of lamotrigine promotes a conformational change characterized by a α→π transition in the middle of the S6IV region and, to a lesser extent, movements of S6II and S6III. Figure 4 shows the superimposition of the I- and BIG sites of NaV1.7, in the presence of CBD or lamotrigine, respectively. The binding sites are contiguous and delimited by the S6IV domain, which changes its position to accommodate these ligands. Some amino acids are shared by the two binding regions. The results obtained indicate conformational changes induced by the presence of CBD in the I site that affect the formation of the BIG site and prevent the interaction with lamotrigine.

**FIGURE 5:**
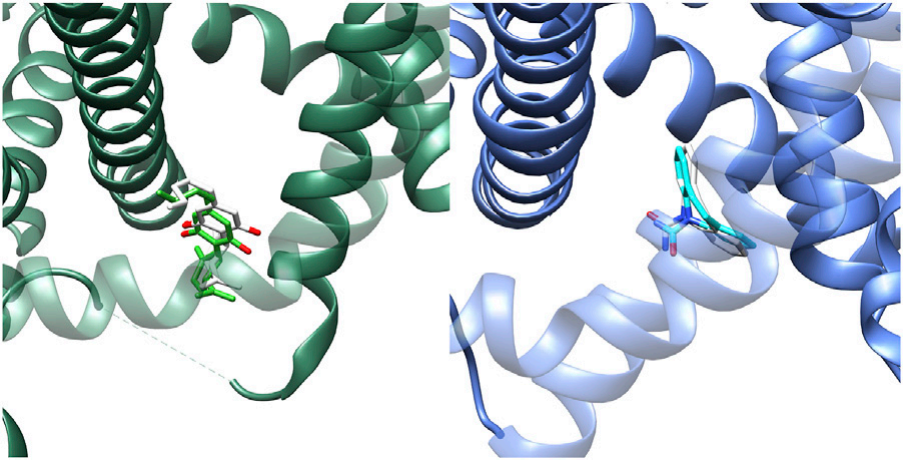
Results obtained from re-docking validations with Autodock Vina. The experimental conformations are depicted in lime green and light blue for CBD (left) and lamotrigine (right) respectively whereas the results of the simulations are highlighted in white.

**FIGURE 6:**
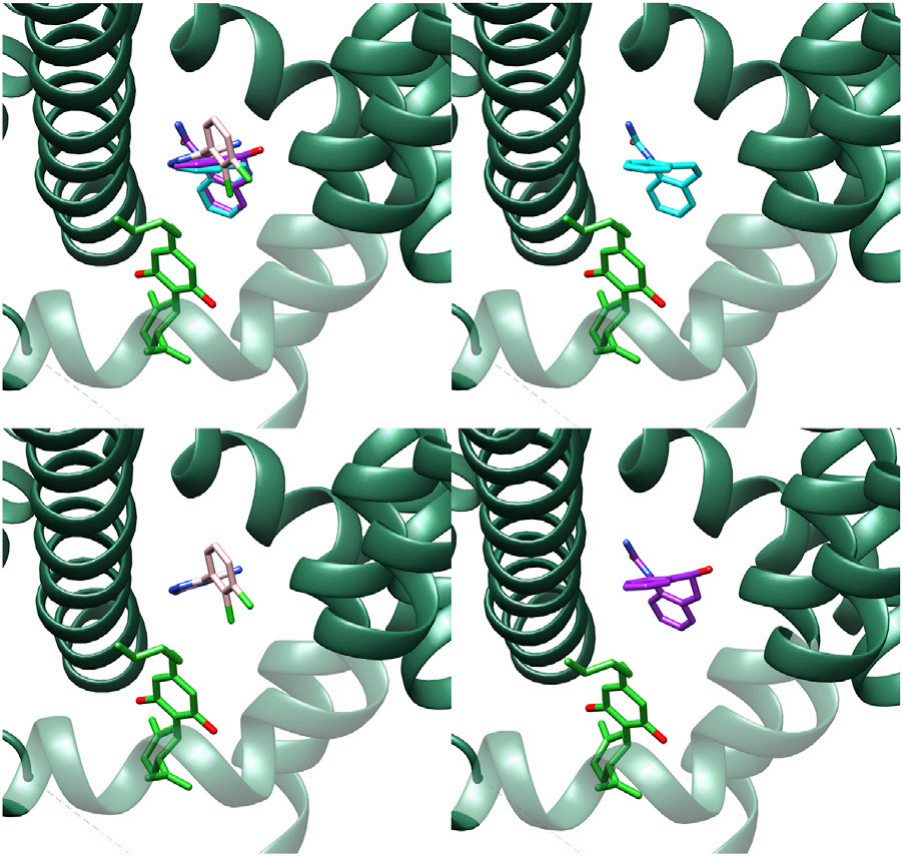
Best poses for carbamazepine (light blue), lamotrigine (pink) and the active metabolite of oxcarbazepine, (S)-(+)-10-Hydroxy-10,11-dihydrocarbamazepine (S-MDH) (purple) in the Nav1.7 experimental structure co-crystallized with CBD (8G1A) obtained with Autodock Vina.

At present, there is no structural data available about the interaction of oxcarbazepine with sodium channels. On the other hand, the high molecular similarity between carbamazepine and oxcarbazepine suggests that both drugs are likely to interact in a similar manner with the sodium channels. Then, we performed docking simulations considering the BIG site, a region in which carbamazepine interacts (Wu et al., 2023). Re-docking validations (Figure 5) showed that Autodock Vina was more accurate to predict the experimental binding pose. Figure 6 shows the binding mode proposed for the active metabolite of oxcarbazepine, (S)-(+)-10-Hydroxy-10,11-dihydrocarbamazepine (S-MDH). Interestingly, docking simulations of lamotrigine and S-MDH into the conformation induced by CBD to NaV1.7 (PDB-ID 8G1A) showed that the binding region of these ASMs is modified upon CBD binding (Figure 5).

## 4 Discussion

The present study confirms that CBD alone does not modify the mortality rate induced by severe seizures (Anderson et al., 2020). Our results also support that CBD combined with GABAergic drugs but not with sodium channel blockers reduces the mortality rate during the repetitive induction of clonic-tonic seizures and prevents the development of drug-resistance seizures in a preclinical model.

The ED_50_ calculated for the different ASMs evaluated in the present study were like those used in previous preclinical models of generalized seizures (Jindal et al., 1968; Shenoy et al., 1982; Moss et al., 1986; Wamil et al., 1994; Chen et al., 2017). The results obtained indicate that the repetitive coadministration of CBD and ASMs at ED_30_ or ED_50_ in control animals does not induce side effects (impairment of motor coordination, changes in neuromuscular strength or CBD-induced hypolocomotion) that can be confused with anticonvulsant effects. Indeed, these findings support that the combinations of CBD and the ASMs evaluated in the present study have a favorable safety profile.

Our experiments revealed that CBD combined with sodium channel blockers does not modify the mortality rate during the repetitive induction of clonic-tonic seizures. In addition, this combination did not prevent the development of drug-resistance phenotype. Indeed, we found that the combination of CBD and oxcarbazepine worsens the drug-resistant phenotype induced by repetitive severe seizures. The absence of interaction between CBD and valproic acid detected in the present study is consistent with the effect of CBD in a preclinical study of audiogenic seizures (Cabral-Pereira et al., 2021).

A previous study suggests that the attenuated anticonvulsant effect of levetiracetam when combined with CBD in the 6 Hz seizure test in mice results from a pharmacodynamic interaction between both drugs (Socała et al., 2019). In the present study, as a first approach to explain the experimental results at molecular level, the structural information available about two binding sites for small molecules in NaV1.7, I-site and BIG site, was analyzed and complemented by validated docking simulations. We propose that the induced fit effect triggered by CBD in the event of binding into the I-site could change the conformation of the BIG site, altering the interactions of the target with lamotrigine and oxcarbazepine. This would partially explain the negative effects found *in vivo* with the coadministration of CBD and either lamotrigine or oxcarbazepine. However, more structural data, *in vitro* assays on the target and pharmacokinetic studies will be necessary to better support this hypothesis.

The tolerance to the anticonvulsant effects and facilitation of the drug-resistant phenotype induced by the chronic administration of sodium channel blockers can also explain the negative results obtained with lamotrigine and oxcarbazepine. The repetitive administration of lamotrigine during the development of electrical kindling results in seizures resistant to this drug, carbamazepine, phenytoin and topiramate (Postma et al., 2000; Srivastava et al., 2013; Koneval et al., 2018). Similarly, the chronic administration of oxcarbazepine reduces its anticonvulsant effect in the maximal electroshock test in mice (Borowicz-Reutt and Banach, 2024). It is suggested that the repetitive administration of some sodium channel blockers may affect the expression of sodium channel subunits, resulting in a faster recovery from the inactivated state (Schmidt and Löscher, 2005; Borowicz-Reutt and Banach, 2024). Additional experiments are essential to support this hypothesis.

Other explanation relies on pharmacokinetic interactions between CBD and some ASMs (Zendulka et al., 2016). CBD potentially interferes with oxcarbazepine metabolism by suppressing its glucuronidation (Stout and Cimino, 2014). This effect can result in high levels of oxcarbazepine, a situation that may facilitate the tolerance to its anticonvulsant effects. On the other hand, it is described that CBD at high concentrations may augment the extracellular release of glutamate in synaptic terminals obtained from patients with drug-resistant epilepsy (Martínez-Aguirre et al., 2023). If oxcarbazepine significantly raises the CBD concentrations in the brain (Socała et al., 2019), the coadministration of both drugs may result in excitatory effects due to enhanced glutamatergic neurotransmission.

The results obtained revealed that the efficacy of phenobarbital or diazepam alone at ED_30_ or ED_50_ to protect the 30% and 50% of animals respectively, from acute severe seizures induced by 3-MP is not maintained during their repetitive administration. This effect can be explained by tolerance induced by the repetitive administration of GABAergic ASMs (Kapur and Macdonald, 1997; Guignet et al., 2024). However, when combined with CBD, repetitive administration of phenobarbital and diazepam have potential disease-modification activity, since they diminished the severity of clonic-tonic seizures and avoided the development of drug-resistance to these ASMs. This is consistent with a previous study in which the administration of CBD augmented the anticonvulsant effect of phenobarbital in a model of severe generalized seizures in rats (Frías-Soria et al., 2021). The enhanced effect of GABAergic drugs when coadministered with CBD can be explained because of its action as an allosteric modulator of GABA_A_ receptors (Bakas et al., 2017).

The present study highlights the relevance to applying appropriate combinations of ASMs and CBD that reduce the mortality rate and development of drug-resistance in clinical disorders such as Dravet syndrome in which only 10% of the patients’ symptoms are controlled with polytherapy (Samanta, 2020). Further studies are essential to determine the effects of CBD combined with different ASMs in patients with specific types of epilepsy. Indeed, our experiments revealed that CBD was able to augment the effect of phenobarbital at ED30 and ED50. However, CBD augmented the efficacy of diazepam only at ED50. These results indicate the necessity to carry out analysis such as isobologram methods to determine the proper interactions between CBD and different ASMs. It is relevant to conduct preclinical pharmacodynamic studies to determine the best interactions between CBD and other ASMs. In addition, changes in the intrinsic severity of seizures during the effects of CBD and ASMs should be assessed by evaluating different parameters such as the frequency and duration of the seizure activity.

## Data Availability

The raw data supporting the conclusions of this article will be made available by the authors, without undue reservation.
